# Human-Centered Design and Digital Transformation of Mental Health Services

**DOI:** 10.2196/66040

**Published:** 2025-08-11

**Authors:** William Fleming, Adam Coutts, Diane Pochard, Daksha Trivedi, Kristy Sanderson

**Affiliations:** 1 Wellbeing Research Centre University of Oxford Oxford United Kingdom; 2 NIHR Applied Research Collaboration East of England University of Cambridge Cambridge United Kingdom; 3 Centre for Science and Policy University of Cambridge Cambridge United Kingdom; 4 Centre for Research in Public Health and Community Care School of Health, Medicine and Life Sciences University of Hertfordshire Hatfield United Kingdom; 5 NIHR Applied Research Collaboration East of England University of East Anglia Norwich United Kingdom

**Keywords:** digital mental health, digital transformation, human-centred design, mental health policy, mental health services, patient and public involvement

## Abstract

Mental health services face a multitude of challenges, such as increasing demand, underfunding, and limited workforce capacity. The accelerated digital transformation of public services is positioned by government, the private sector, and some academic researchers as the solution. Alongside this, human-centered design has emerged as a guiding paradigm for this transformation to ensure user needs are met. We define what digital transformation and human-centered design are, how they are implemented in the UK policy context, and their role within the evolving delivery of mental health services. The involvement of one of our coauthors (DP) in the design and delivery of these policies over the past 5 years provides unique insights into the decision-making process and policy story. We review the promises, pitfalls, and ongoing challenges identified across a multidisciplinary literature. Finally, we propose future research questions and policy options to ensure that services are designed and delivered to meet the mental health needs of the population.

## Introduction

### Background

Mental health services are under strain in the United Kingdom [1,2]. Demands for assessment, diagnosis, and treatment are increasing [3], while funding and infrastructure struggle to keep up and are widely seen to be in crisis [4]. Waiting lists and prolonged referral times are commonplace, with disastrous consequences for the well-being of service users [5]. Frontline organizations struggle to recruit, retain, and train staff, with the mental health of those in position already at risk [6,7]. The United Kingdom and others across Europe and the Organisation for Economic Cooperation Development countries are seeking innovative and ambitious policy solutions [8].

Digital transformation is widely touted as the answer to these challenges, promising affordability, accessibility, and rapid scalability [9,10]. In the United Kingdom, widely regarded as a leader in digital transformation across policy domains [11,12], a digital-first delivery model is now embedded in the long-term policy for the health service [4,13,14]. Special attention is placed on the expansion and reform of mental health care [15,16], with a view to supporting financial cuts. Globally, the digital delivery of mental health services is viewed as vital to meet demand [17-20], especially for the poorest and the hardest-to-reach populations, whether culturally, geographically, or economically [21,22].

Despite national and international enthusiasm from policy constituencies and academic researchers, digital transformation remains an ambiguous concept [23] and is poorly defined on the ground. Digital transformation has been ongoing in health care for decades as various information and communication technologies (eg, emails, mobile phones, and electronic health records) were integrated into service delivery, at times through professional and patient discretion. Still, recent calls for acceleration and increased policy discourse demand a robust definition moving forward. Furthermore, despite optimism, digital transformation risks being an inequitable and ineffective process if it fails to meet the needs and requirements of service users, health care providers, and professionals [24]. In response, service-user involvement is increasingly advocated in the form of human-centered design (HCD) [17,25,26].

### Outline

We begin by defining digital transformation and HCD for mental health services. We contextualize these ideas and processes in a personal view section, including a case study of the Mental Health Act (MHA) in UK policy. These policy insights draw directly from our experiences implementing HCD digital transformation in the UK Department for Health and Social Care (DHSC).

In the second half of the paper, we integrate academic knowledge on these themes, bringing researchers and practitioners together on policy and research developments. We first discuss opportunities and challenges in digital therapeutics before emphasizing the need for service-user involvement in therapeutics. We then move to digital transformation of services more broadly. We argue that HCD is essential to the digital transformation of mental health. In so doing, we warn against technosolutionism in digital transformation [17]. Finally, we summarize ongoing service challenges, avenues for future research, and policy recommendations. Overall, the paper contributes to an ongoing debate on the digital transformation of essential public services.

This review is a product of a National Institute for Health and Care Research Applied Research Collaboration East of England project, which holds knowledge exchange to be central. We brought together researchers, service delivery experts in the DHSC, the National Health Service (NHS), the Office for Health Improvement and Disparities*,* as well as local coordinators and frontline staff in the east of England. The review is informed by the ongoing exchange, including interviews with practitioners and a training workshop we hosted on “human-centered design for mental health services” [27]. Among frontline professionals we found enthusiasm for HCD, alongside many practical constraints to implementation. The search strategy and selection criteria are as follows: we identified relevant articles in English through structured searches on Web of Science and unstructured searches on Google Scholar using key terms, such as “mental health,” “human-centered design,” “co-production,” “digital,” and “services.” Our disciplinary focus was journals in psychiatry and public health as well as relevant literature from public policy and design. We also searched the key terms in *The Lancet Psychiatry*, the *Journal of Medical Internet Research,* and *JMIR Mental Health*. Relevant policy gray literature is included to define the context, sourced from professional networks, and project stakeholder engagement.

## Defining Digital Transformation

Digital transformation is a poorly defined concept, with multiple uses found across practitioner and academic literature. For example, the UK DHSC does not offer any definition in their policy paper “A plan for digital health and social care” [13], despite central claims that “the long-term sustainability of health and social care is dependent on having the right digital foundations in place, and so digital transformation must be the linchpin upon which all of these reforms are based.” Ambiguity is repeated in government guidance for UK NHS trusts [28] and in global settings, with the World Health Organization neglecting to define digital transformation, despite its “Global strategy on digital health 2020-2025” [18].

In a systematic review of the digital transformation literature, Vial [23] offers a synthesized definition: “a process that aims to improve an entity by triggering significant changes to its properties through combinations of information, computing, communication, and connectivity technologies.” In the context of increased discourse around the digitalization of mental health and the need to understand implementation and outcomes, a domain-specific definition of digital transformation is required. We adapt and define mental health digital transformation as “a process that aims to improve the provision of mental health services by triggering significant changes to its administration, coordination, treatments and therapeutics, through combinations of information, computing, communication, and connectivity technologies.”

## Defining HCD

The concept of HCD originated in the fields of computer science, engineering, and ergonomics. The technical origin is captured in the International Organization for Standardization definition of HCD [29], but the emphasis in early technology-oriented definitions centers the relationship between individual users and physical objects. The conceptual scope of HCD expanded as the social potential was realized: “today’s human centred design is based on the use of techniques which communicate, interact, empathise and stimulate people involved, obtaining an understanding of their needs, desires and experiences” [30]. HCD principles can guide value and well-being creation in the delivery of products, systems, and services. We define HCD in mental health services and research as “a practical, iterative approach to the design, development and reform of mental health systems, services and products that is achieved through communication, interaction and empathy with users’ needs, desires and experiences.”

**Figure 1:**
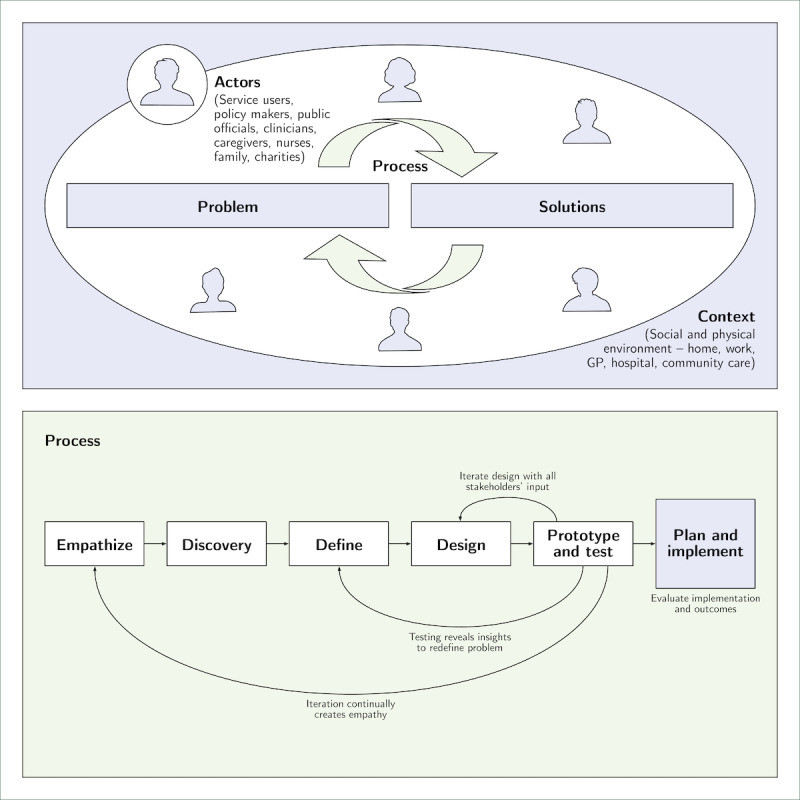
Human-centered design process for mental health services. GP: general practitioner.

Figure 1 summarizes the HCD approach. HCD is applied in an iterative process and includes 4 dimensions: the problems and coevolving solutions (*objects*), social and physical environment (*context*), structure and dynamic of the activity (*process*), and those involved (*actors*) [30,31]. In the process, HCD deploys a range of qualitative techniques, such as service logic modeling, semistructured interviews, focus groups, and ethnographic research. This qualitative view creates *empathy* in the *discovery* phase to *define* the issue, including identifying user needs, the interdependencies and interactions between different user groups, and how users navigate and interact with services and products. The aim is to understand the overall context and provide a map of pathways through which a service or product user moves. The new qualitative understanding then guides the *design* of the product and service to be *tested* iteratively in a prototype phase, continuing to redefine the issue and provide new insight. Final *implementation* is enacted while quantitatively evaluating processes and outcomes.

Accompanying the expansion of HCD is increasing conceptual confusion, with related terms such as user-centered design, user experience design, stakeholder-centered design, design thinking, and participatory design [17,31-33]. HCD is the most theoretically developed and commonly applied in policy. We propose HCD can be used as an umbrella term for iterative end user involvement in product and service delivery that emphasizes empathy, discovery, and testing. Synonymous terms can be understood as types of HCD, deployed in different contexts with varying aims.

Coproduction is a crucial related idea that dominates discussion of user involvement in public services. Coproduction is a broader umbrella concept that also entails citizen and user involvement to varying degrees [34-37]. In contrast to HCD, coproduction emphasizes sharing power (decision-making) and collaboratively delivering a service or product. With HCD, decision-making and delivery remain with the service or product provider while strongly involving user input in the iterative process outlined above. HCD is a lighter approach to citizen involvement. Figure 1 visualizes the HCD process and demonstrates the underlying principles, which are more important than semantic debates [37,38].

## Personal View: Untold Stories From the United Kingdom

### Overview

In the United Kingdom health care system, digital transformation methodologies were formally enacted with the creation of “NHSX” [39]. However, the origins and design of the “whole of government” approach were first articulated by the review by Lane Fox [40]. The report emphasized the need for a user-centered digital transformation and a policy culture that is “putting the needs of citizens ahead of those of departments” [40].

The Government Digital Service (GDS) unit within the Cabinet Office was created with the mandate to digitally transform the whole of government and public services more broadly, while introducing new ways of working, such as agile delivery and HCD [41]. While GDS standards, resources, and practices offered a baseline, fragmented governance, dispersed responsibilities, and split accountability among multiple agencies in the health care system often led to inefficient transformation and processes losing sight of the end user.

NHSX was embedded with the mandate of user-centered design, with “X” standing for “user experience.” NHSX was the convening body between DHSC (which designs policy), NHS England (which currently designs policy implementation but is soon to be subsumed in DHSC), and NHS Digital (which delivers technology), alongside arm’s-length bodies of the NHS [42]. In the June 2021 health ministerial transition, an independent review was conducted to assess progress in digital transformation, how to build on what had already been delivered under NHSX’s mandate, and to inform future structuring and investment decisions for NHSX as well as its consequences for other NHS institutions [43]. Therefore, NHSX was dissolved, and a new transformation directorate was created, hosted by NHS England.

### Digitizing the MHA

An example of an “untold story” [44] on an NHSX project is the application of HCD and digital transformation to reforming the MHA. The HCD discovery phase was conducted internally in the UK government and until now has not featured publicly in academic or policy debate.

The MHA is the main legislation covering the assessment, treatment, and rights of people with mental health disorders. As the review of the MHA was being commissioned, the intention was to “make sure those suffering from mental health issues are treated with dignity and respect, with their liberty and autonomy respected” [45,46]. MHA reform began following recommendations arising from another independent review, “Modernising the Mental Health Act” [47]. The recommendations, accepted by the DHSC [48], were to modernize the act, digitize processes, and design a more human-centered system, ensuring “a person’s wishes and preferences will have much more impact and be harder to ignore than before” (47: 186).

DHSC commissioned and delivered a series of discoveries on the act. Discovery is the first qualitative stage of exploratory work, outlined earlier, to identify problems and user needs. One area of focus was advance choice documents (ACDs) [49], which allow a person to set out preferences on future care and treatment, especially in cases of detention. This exploratory work examined the various service pathways people experience the application of the MHA, such as the psychological state that people are in at engagement and where “pinch points” are for service users and staff. Through HCD methods, different users of the ACD system were identified [49]. Discovery primarily involved semistructured interviews with these users, including service users or those at risk of detention, their support network, approved mental health professionals, responsible clinicians, charge nurses, and care coordinators. Exploration also included questionnaires, secondary research, and further focus groups with mental health professionals. The findings provided several recommendations and insights for all types of users.

From the user research conducted, we learned that in moments of acute crisis, there is often no time to meaningfully incorporate patient preferences. People with lived experience described feeling that the most distressing part of being detained is the absence of agency in decision-making. They expressed the need to feel listened to, treated with respect, and “not just a number.” From professionals’ perspective, user involvement emphasized the importance of understanding why a patient would or would not seek specific treatments. Approved mental health professionals we spoke to also expressed concerns about the practical limitations they face in accessing up-to-date medical information and about the crucial challenge of ensuring ACDs reflect the most up-to-date picture of a patient’s needs and circumstances. Nurses also shared concerns about how staff-to-patient ratios directly affect the ability to engage in person-centered care.

Further noted challenges were the lack of information interoperability between care coordinators’ systems and external health care professionals. Overall, the process was felt to have created empathy and illuminated implementation barriers as well as specific challenges and contexts from different users’ perspectives. This HCD process emphasized the crucial role of human relationships in improving care, rather than specific needs for digital services and tools. The follow-up recommendations from the study were to test prototypes for ACDs through a pilot program designed to understand uptake, ease-of-use, and effectiveness across a range of roles and to identify challenges and improvements. Unfortunately, government prioritization decisions meant that recommendations were not put into action.

## Digital Mental Health Research

### Digital Therapeutics

In most discussions of digital mental health, the emphasis is on “apps, social media, chatbots, and virtual reality” [50]. Digital interventions like these are seen as the solution for growing demands for mental health support. Benefits appear to be affordability, accessibility, quick response times, potential integration of resources, and creation of large amounts of data [26,50-53]. The largest benefits are expected for young people who are digitally native [50,51], underrepresented racial and ethnic minority groups [54-56], and low- to middle-income countries where mental health care is minimal to absent [21]. Digital interventions are viewed as especially helpful during acute personal crisis such as suicide prevention [56,57], and external crisis, such as pandemics and natural disasters [58-60]. Researchers are generally positive about the promise of these digital therapeutics [57,61,62], especially through self-help [57,63-65]. However, there are difficulties for the practical widespread adoption, with several reviewers unwilling to recommend adoption and expansion of digital interventions, judging the current evidence inadequate [66,67]. Claims that digital transformation will address health inequalities are also yet to be substantiated, with existing evidence for health care broadly indicating they can exacerbate inequalities through unequal access and outcomes [24].

There are further difficulties for digital interventions. There are a vast number of digital mental health tools, with, for example, estimates of over 15,000 mobile mental health apps [68,69]. Therefore, continued use of the apps is low, with high attrition [68,70,71]. Preventing uptake and upkeep are concerns around data transparency, lack of regulation, and inadequate links with existing services for both patients and clinicians [50,53,69,71]. There is a polarization between low-use, evidence-based practices and high-use, less substantial platforms, accelerated by overcommercialization reducing the quality of products [72]. Furthermore, transplanting digital interventions from trial settings into real-world contexts is a major hurdle [73]. Other difficulties are known too, such as little engagement from those with severe mental illness [74], supply-side technical issues, and a lack of personalization [75]. Clinician involvement in design and implementation is found to produce better effects, but this is not always practiced [76].

Some of these problems arise from limitations in underlying research, resulting in researchers, practitioners, and policy makers being unable to make clear judgments. Trials often target less complex mental illnesses with more standardized solutions, such as generalized anxiety disorders and cognitive behavioral therapy (CBT), acceptance and commitment therapy, and mindfulness [61]. Witt et al [67] state that “most studies are biased” and “it is unclear whether reductions would be clinically meaningful.”

As one of the more controversial examples, chatbot “conversational agent” therapies can exemplify the difficulties. Chatbots are one artificial intelligence–driven therapy enthusiastically proposed as cheap and effective [77]. Reviewers have argued that trials are of low-quality study design overall, with small samples, high attrition, negligible effects, and a high risk of bias [78]. However, new evidence is frequently published, pointing to the benefits of generative artificial intelligence therapeutic solutions for clinical depression [79]. Further user involvement is needed for assessing efficacy [17].

While chatbots exemplify the need for user engagement where therapeutic efficacy is not assured, the same is cited for proven digital interventions. Togetherall is an illuminating example of a prominent peer support platform for mental health [80,81]. Across multiple studies, partnered researchers show that service can address gaps in provision, offer support for those on waiting lists, and provide self-management opportunities. These studies indicate positive signs but reveal an ongoing need for rigorous and independent evaluation.

### HCD and the Need for User Involvement

As in policy reviews, academic commentators continually call for more user involvement in the design and implementation of digital interventions [25,26,51,82], proposing coproduction as the “standard way of working” in digital mental health research [83]. This desire is echoed by practitioners and patients as well [84], all recognizing the need for co-designed solutions for effective and ethical care [85]. Therefore, it should not be surprising that HCD emerged as a guiding paradigm alongside digital transformation. Indeed, commentators in the design literature cite the digital transformation of public services as the reason why HCD has become so popular [86,87].

Vial et al [31] review the prevalence of HCD principles in the development of digital mental health interventions. They find 22 studies mentioning HCD or specific design methods, offering some promise for the integration of these ideas into digital mental health products. Since then, other interesting case studies have been published. The best example is perhaps from Creswell et al [88]. The “digitally augmented” platform they co-designed with users [89,90] provides online training and therapist support for parents to deliver themselves in-person CBT treatment for their child’s anxiety. They find this parent-led, real-world application to be as effective as regular therapist-delivered CBT and more cost-effective. The strengths of the intervention stem from their HCD approach, ensuring fit with all user needs and professional support throughout. Our examples show that digital technologies can support the improvement and expansion of mental health services, with HCD principles enhancing development to meet service-user needs. These results suggest that social interactions remain key to effective treatment, while people can be supported with digital tools in the delivery rather than replaced.

### Digital Mental Health Services

### Overview

Within digital mental health research and policy, there is an overwhelming focus on the delivery of therapeutic interventions. This can be seen across many of the systematic reviews [63] and policy documents on the topic. However, this limits the potential of digital transformation and hinders the stated aim of improving the effectiveness, affordability, and scalability of mental health services for all. Further uses are evident in the studies by Naslund et al [91] and Stein et al [92].

### Data Collection and Sharing

Digital technologies offer opportunities for the collection, communication, and analysis of patient data. Internal electronic health records are an early example of digital transformation in health services. A more recent patient-focused avenue is patient-accessible electronic health records (PAEHRs). In recent years, health services in North America and Europe have allowed patients to view health data and clinical notes (eg, the NHS App). The aim is to improve transparency, patient engagement, patient-clinician communication, and health literacy [93,94]. Service-user involvement in the design of PAEHRs shows that different mental illnesses reveal different attitudes toward the practice. Those with more severe and chronic mental concerns decline open PAEHRs more often and are more critical of systems, and in cases of severe mental illness, records are often concealed [95]. Systems can be more equitable with HCD applied in the development and ongoing improvement of services [94,95].

Linkage and interoperability are ongoing data challenges, highlighted in our discussion of the MHA [96,97]. Data sharing is vital for the delivery of services, with overlapping policy domains of health, employment, housing, and education. In our stakeholder engagement we learned of difficulties faced by asylum-seekers and refugees in the United Kingdom who would be “bounced” between immigration, housing, health, and employment systems with minimal communication or knowledge sharing between organizations [27]. Considered adoption of HCD could develop these insights for improving information sharing.

### Assessment

Assessment and diagnostics appear fruitful for the implementation of digital technologies [51]. Multiple studies propose digital screening in general practice offices to identify mental health issues [98,99], alleviating resource constraints on professionals. While promising, there have been too few attempts to seriously involve those being assessed in the design of digital screening tools. One exception is the depression monitor by Nickels et al [100], which correlated electronic pulse surveys with smartphone data to explore its applicability in place of targeted questionnaires. Another radical form of assessment is the use of smart home sensing by Tiersen et al [101] for those with dementia, finding HCD enhanced applicability and relevance.

### Promotion and Primary Prevention

Mental health promotion is frequently proposed as a fruitful domain for digital transformation [10,17,102-104]. Assessment and diagnostics is a key component of prevention to develop early intervention, but promotion and prevention can take on a variety of forms. Some interventions include education (to follow), mood management, diaries, peer support, or community engagement. Some level of co-design is almost always argued in the development of prevention interventions to ensure either underrepresented voices are included [103], engagement with products is maintained [102,104], or there is trust generally between users, designers, and health professionals [102].

### Education

Digital transformation and HCD can come together on a smaller scale than full system change. Education is one such area where research shows digitalization can assist and be effectively designed for information provision for either clinicians or citizens [105,106]. For example, Manning et al [107] show a digital training program for nurses can improve professional effectiveness when co-designed by young people who have self-harmed. Digital learning can also benefit communities through mental health literacy, especially in contexts with previous low levels [108].

### Rehabilitation

Finally, digital technologies may help with the rehabilitation process. Engdahl et al [109] show that their co-designed, digital return-to-work scheme is an effective way of supporting those with common mental illnesses. “mWorks” operates as a supplementary self-management tool for existing return-to-work services to prevent relapse. The participatory aspects were central to the success of the platform. Such an initiative shows that successful digital mental health tools may supplement rather than replace core services.

### “The Solution May or May Not Be Digital”: HCD in Mental Health Services

We have highlighted areas that indicate benefits from HCD in digital mental health. However, by only considering HCD principles in the design of digital therapeutic tools, researchers, practitioners, and policy makers would fail to embody the ideals of evolving services to meet citizen needs. HCD promotes solutions tailored to user needs discovered through qualitative engagement. It may be that appropriate solutions are not digital but instead other service reforms or improved financial, staffing, and technical resources. An implicit “technosolutionism” [17] in discussions of HCD in psychiatry can be seen by the emphasis placed on using it to enhance acceptability of these technologies [110]. Similarly, assuming digital transformation will aid co-creation methods risks ignoring that they may instead be used to bypass democratic oversight and citizen involvement [111]. These contradictions demand a more thorough integration of HCD principles into the design of mental health services as a whole.

HCD appears beneficial for the reform of health services—digital or not digital [87,112]—while lessons can be learned from the previous application of HCD in the public sector outside of mental health. HCD and coproduction are proposed by public policy scholars as ways to create public value in services, enhance trust in the face of democratic deficit, create active citizens and communities, and enhance the legitimacy and communication of science [35,36]. Bason and Austin [113] review various service and policy redesigns in areas such as family services, housing, and education, finding multiple advantages in HCD projects, including the ability to identify previously unknown barriers and iteratively improve services. They suggest HCD can produce a model of public governance that is more relational, networked, and reflexive. Several commentators outline these same empowering benefits in health and care services but cite several challenges for embedding HCD as best practice [114,115].

The value of HCD for service delivery is demonstrated beyond academic research teams. One case highlighted through stakeholder engagement was conducted by the mental health charity, Mind. They co-designed perinatal support for Muslim women in the east of England [116], a population that is typically considered difficult to reach. Mind also publicly detailing their commitments to human-centered service design and offering guidance for others who are interested in these methods [117]. The charity Mind shows that HCD is relevant for third-sector actors as well as for public policy, academics, or commercial developers.

## Ongoing Challenges: Directions for Research and Policy

### Research

Digital transformation and HCD ideals can offer great potential for expanding and improving mental health services. However, we see several ongoing challenges for researchers, practitioners, policy makers, and service users.

First, many of the ideas within HCD principles are not new to health care delivery, with elements found in, for instance, “no decision about me, without me” [118], patient and public involvement, triangle of care, and person-centered care. HCD brings with it structured and working methodologies as well as more expansive considerations, but progress from previous citizen involvement agendas must not be forgotten or lost amid calls for the latest trending policy paradigm.

As discussed, technosolutionism can limit the impact of HCD if digital transformation is assumed to be the required resource for meeting needs. Next, a common concern in HCD and coproduction is continued power asymmetries [119,120]. For many, “the gap between principles and practice is challenging” [121], with dangers of practitioners, researchers, and designers acting “as if” power boundaries are blurred [122], when in reality the resources of current and future users of service are not enhanced [123]. Relatedly, service-user involvement must cover underrepresented populations [124]. This is most likely to be those with more severe mental conditions, exposed to multiple deprivations, and from discriminated groups. Difficulties, including the hardest-to-reach populations, emphasize that HCD will not guarantee success in the delivery of mental health services.

In addition, there are tensions between medical models of health care and the person, patient, or citizen emphasis of HCD. Traditional models of health care assume that expertise lies with professionals who have extensive training and clinical experience. Indeed, this is especially relevant in severe cases of mental illness; for example, following our discussion of the MHA, a person may be detained against their wishes. Mental health care generally requires classification and standardized protocols such as the MHA. In contrast, HCD assumes that the user is the expert [125], and in the case of mental health, where the experiences are intensely subjective and often context-specific, it poses domain-specific difficulties for developing universally or nationally appropriate services. This epistemic and political incompatibility is a common challenge elsewhere in coproduction, leading to the ideal of coproduction being undermined, proving tokenistic or “pretend” [122,126,127]. In HCD, where the ultimate delivery remains with the service provider, these limitations are less crucial. Service provision can be designed with strong involvement of service users, without appearing to dispense with professional health expertise. Nevertheless, as with all involvement practices, a central challenge remains which is achieving successful, meaningful engagement with service users [126,128,129].

To evaluate these challenges, HCD digital transformation must embed implementation evaluation and success metrics from the start and include user involvement in this design process [31,68,130]. This includes developing theories of change [81,131], identifying process mechanisms, and safeguarding those who may be negatively impacted by interventions [67].

Finally, thorough HCD can be time intensive, requires staffing commitments and funding, and can be difficult to standardize [131]. New policy structures, regulatory and commissioning practices, and funding are needed if HCD is to be a long-term solution [115,131], especially when facing the need for cost-effectiveness of digital solutions. Recent regulation of digital mental health technology from government agencies is a welcome protection against these latter harms [132]. In our own stakeholder engagement, the cost and time for co-designing were cited as limitations, but so too was the feeling that these ideas and digitalization were a top-down policy approach putting pressure on service delivery instead of providing funding. We learned of the gaps between, on one hand, those designing policy plans for digital transformation and ideals of HCD, and on the other, the realities of underresourced services. The future research agenda is given in Textbox 1.

**Future research agenda.**

- How effective is large-scale digital transformation for meeting service-user needs and health outcomes?
- What are the barriers to engagement in human-centered design (HCD) and coproduction processes?
- How can user involvement be better integrated into data sharing, linking, and interoperability systems?
- How are government digital transformation policy mandates received by practitioners in health service delivery and local authorities? How are HCD ideas translated and received across levels of governance?
- How does prospective regulation of artificial intelligence and other emergent technologies link to digital mental health?
- What is the political economy of digital transformation and HCD in mental health services, including political and economic incentives at multiple levels, funding arrangements, and private capital involvement?
- Where do “policy ideas” on mental health emerge from?

### Policy Recommendations

#### Overview

Our review of the literature reveals many opportunities, but also challenges, for the digital transformation of mental health services, and especially the contradictory promise of HCD. We urge caution on technosolutionism, with increased institutional support and attention to the tensions between technocratic and HCD models as well as implementation and evaluation.

Similarly, insights from various NHSX projects, in the personal view section, reveal systemic issues that hinder the design and provision of HCD: the absence of agency for patients, staff shortages, operational pressures that prioritize risk-based approaches, fractured data systems, lack of systems’ interoperability, and outdated data and digital infrastructure. These realities highlight the need for structural, policy, and capability reforms that go beyond individual behavior change to reshape decision-making systems.

The recent political decision to abolish NHS England marks a significant shift in UK health care, offering a unique opportunity to redesign the system for greater efficiency and responsiveness. The elimination of duplicative governance structures and clearer decision-making pathways bring modernization possibilities for operations and realignment with user needs.

Therefore, in conclusion, we advance several policy recommendations for mental health in the United Kingdom, developed from our review, stakeholder engagement, and professional experience of digital transformation and HCD.

#### Independent and Cross-Departmental Digital Transformation Commission

The digital transformation directorate currently within NHS England should be externalized to establish an autonomous unit tasked with leading digital transformation across the health care ecosystem. A common strength of GDS and NHSX lies in their external positioning vis-à-vis “existing routines” and political constraints [11]. An external directorate would provide more effective convening powers, enabling better collaboration and system-wide alignment of incentives, efforts, and decisions across national and local public institutions and arm’s-length bodies.

#### Agreed Definitions of Methodologies and Outcomes

In the course of this paper, we have developed definitions of digital transformation and HCD with respect to mental health services, and these should be incorporated into the digital transformation directorate. We hope and intend for these to be used in future policy work. Further research is needed to agree on the criteria for successful transformation, standardized processes, cost guidelines, capability assessment and needs guidance, and impact evaluation methods.

#### Digital Standards Collaboratively Defined

Similarly to the first recommendation, HCD approaches should be leveraged to develop data standards and operability with collaboration across systems for common terminologies, protocols, and exchange standards, as recommended by DHSC [133] and NHSX [134]. Standards and operability must function across policy domains and government departments, linking NHS mental health services with other government departments.

#### Integrate HCD in Other Public Policy Domains and at Both Local and National Levels in Health Services

Health care is viewed as the most promising and important avenue for the adoption of HCD principles [112,135]. Still, HCD and coproduction have been shown to be beneficial in an array of policy settings [36,113]. To facilitate the success of HCD in mental health, extending the application of HCD across public services and at multiple policy levels will normalize and strengthen the relevant social and operational systems required for success. Widespread use of HCD can bring standardization and capacity building and connect it to other models of involvement to ensure meaningful service-user engagement.

## Abbreviations

ACD: advance choice document
CBT: cognitive behavioral therapy
DHSC: Department for Health and Social Care
GDS: Government Digital Service
HCD: human-centered design
MHA: Mental Health Act
NHS: National Health Service
PAEHR: patient-accessible electronic health record
